# Prevalence and Determinants of Severe Mental Disorders in Iran: Evidence from the National Survey of STEPs- 2016

**DOI:** 10.34172/aim.2022.76

**Published:** 2022-07-01

**Authors:** Farshad Farzadfar, Rostam Zalvand, Badrye Karami, Moein Yoosefi, Amirhossein Takian, Maryam Tajvar

**Affiliations:** ^1^Non–Communicable Diseases Research Center, Endocrinology and Metabolism Population Sciences Institute, Tehran University of Medical Sciences, Tehran, Iran; ^2^Department of Health Management, Policy and Economics, School of Public Health, Tehran University of Medical Sciences, Tehran, Iran; ^3^Department of Global Health and Public Policy, School of Public Health, Tehran University of Medical Sciences, Tehran, Iran; ^4^Health Equity Research Center (HERC), Tehran University of Medical Sciences, Tehran, Iran

**Keywords:** Health, Lifestyle, Mental disorders, Severe angry, Severe sadness, Severe stress

## Abstract

**Background:**

The determinants and correlates of severe mental disorders are less understood compared to the common mental disorders, both in the world and in Iran. In this study, we aimed to identify a wide range of determinants of severe stress, severe anger, and severe sadness among Iranian population.

**Methods:**

This study is part of a large nationwide cross-sectional survey entitled STEPs conducted using a comprehensive questionnaire to determine the prevalence of main preventable risk factors of non-communicable diseases (NCDs) in Iran by age and sex groups in 2016. In total, 30541 people aged 18+participated in this study. Univariate and multivariate logistic regression analyses were used to examine the associations between the dependent variable, which is severe mental disorders, and independent variables including socio-economic factors, lifestyle and selected NCDs.

**Results:**

The prevalence of severe stress, severe anger and sever sadness in the Iranian society was 33%, 35%, and 25%, respectively. Of the investigated socio-economic factors, being men, older, never married and living in rural areas were associated with significantly lower experience of severe mental disorders compared to other groups. For education, income and wealth index, there was no linear and clear pattern. Among lifestyle factors, being nonsmoker, having low physical activities, and higher intake of fruits and vegetables were found to be preventive of severe mental disorders. Additionally, having NCDs including hypertension, high cholesterol, diabetes and heart attacks were also significantly correlated with severe mental disorders.

**Conclusion:**

determining factors associated with severe mental disorders in this study would help in raising people’s awareness on avoiding harmful factors, and taking healthier lifestyle such as quitting smoke, and consuming enough vegetables and fruits. Screening high risk people in terms of mental health could contribute to the reduction of mental disorders in the Iranian community.

## Introduction

 Mental health is defined by the World Health Organization (WHO) as “a state of well-being in which every individual realizes his or her own potential, can cope with the normal stresses of life, can work productively and fruitfully, and is able to make a contribution to her or his community”.^1^ Mental disorder is one of the world’s health priorities and constitutes the 3^rd^ goal of the Sustainable Development Goals (SDGs).^2^ According to the WHO, in 2015 nearly 320 000 people died as a result of mental disorders and substance abuse, of which 74% were men and 26% were women.^3^ In addition, the result of a global systematic review and meta-analysis indicated that approximately one out of five persons experienced a common mental health disorder annually during 1980-2013.^4^ Based on the statistics provided by the Global Burden of Disease (GBD) website, depression and anxiety are the most prevalent types of mental disorders, which globally comprises 2.8% of the total disability adjusted life years (DALYs) in both sexes and all ages in 2017, and surprisingly the prevalence rate is more than 2.5 times higher among high-income compared to low-income countries (4.2% vs. 1.6%).^5^ In Iran, the result of the fourth national mental health survey in the adult population in 2015 indicated that 23.5% of people were suspected to have at least one type of mental disorders, which is considerably higher than the world average.^6^ Based on the third national survey, the prevalence of mental disorders increased from 17% in 2001 (since the second survey) to 22% in 2011.^7,8^ Analysis of the national mental health surveys showed that the trend of mental disorders among people aged 18 years and above has increased during 1990 to 2015.^9^ Thus, although mental health is a global issue, this problem is even more significant in the Iranian context.

 To control this problem and improve the mental health status in the Iranian community, initially understanding the role of various determinants of mental disorders is crucial. Many studies, including systematic reviews, have already been conducted in the world,^10-13^ low- and low-middle income countries^14^ and in Iran^6,15-17^ to examine the factors associated with mental disorders. However, most of these studies considered common mental disorders rather than specific or severe mental disorders, or examined the effects of selected factors on these mental disorders.^6,18-20^ Thus, the determinants and correlates of severe mental disorders, both in the world and in Iran, are less investigated.

 In this study, which is based on a national-wide survey data (STEPs), we examined the correlation between three sets of factors including a long list of demographic & socioeconomic factors, lifestyle factors and selected non-communicable diseases (NCDs) with severe mental disorders including severe stress, severe angry and severe sadness. Severe mental disorders, in addition to association with higher rates of mortality, suicide and serious health outcomes for the persons themselves, may have significant negative social and emotional costs for the society, making them much more essential for consideration compared to common mental disorders.^21^ Chronic anger and stress may also reduce intimacy within personal relationships; partners and other family members tend to be more guarded and less able to relax in their interactions with hostile people.^22^ Additionally, uncontrolled anger, stress and sadness may result in loss of employment, loss of one’s family, and even imprisonment.^23^

 Documentation of information on the determinants of severe mental problems can help to identify areas of socioeconomic inequity and serves as a barometer of a society’s health system. This study is conducted with the hope of providing sound and comprehensive information on the determinants of severe mental disorders in Iran among adult population. Providing such evidence would be critically important for effective health policymaking and resource allocation to control the problem, to underpin advocacy efforts, and to stimulate further research.

## Materials and Methods

###  Study Design

 This is a secondary study, performed based on the primary data from the STEPs study. The WHO STEPwise approach to risk factor surveillance, the so–called STEPs, provides a simple, standardized method for collecting, analyzing and disseminating relevant information and helps countries to build and strengthen their capacity for conducting surveillance.^24^ Iran’s STEPs is a large-scale cross-sectional population-based survey of Surveillance of Risk Factors of NCDs in Iran, which is conducted by the NCDs Research Center of the Endocrinology and Metabolism Research Institute in 2016. Study populations were adults aged 18 and over (25-64 for biochemical measures). Using proportionate to size cluster random sampling method, 31050 participants were enrolled from all provinces of the country. Samples were taken from urban and rural areas of 389 out of 429 districts. After applying weight to the samples, comparing the distribution of population and samples, compared classification was made based on the age and sex groups. Of 31050 expected participants, 30541 people finally participated (52.31% female). A comprehensive questionnaire was designed and piloted to collect survey data in order to determining the prevalence of the main preventable risk factors of the NCDs in Iran (including smoking, poor nutrition, low physical activity, high blood pressure, overweight and obesity, high blood glucose, and high blood lipids) by age and sex groups. As a large scale national survey, the feasibility of conducting was evaluated in association with implementation on a national–level scale.^25^

###  Measurement of Study Variables 

 Study variables used to measure severe mental disorders were three single questions of the experience of any condition of severe stress, severe anger, and severe sadness during the week prior to participation in the study. Factors, whose their association with the measures of severe mental disorders were examined, included socio-demographic characteristics (age, wealth index, education, area of living, marital status, occupation, insurance, and income), lifestyle and behavioral information (nutrition, smoking and alcohol consumption, serving of fruits and vegetables, and physical activities), and chronic health status factors (diabetes, hypertension, cholesterol, heart attack, and ischemic stroke).

###  Statistical Analysis 

**Figure 1:**
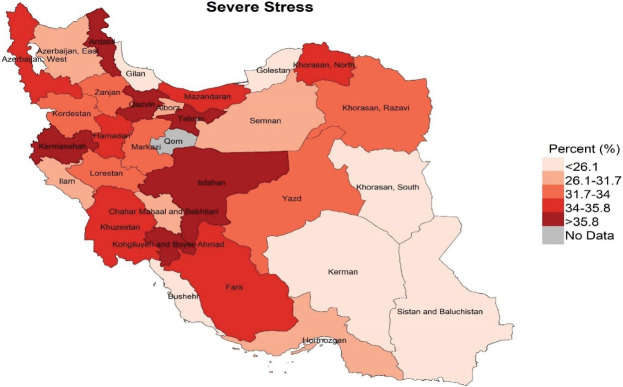
Distribution of Severe Stress among People Aged 18 Years and Over in the Provinces of Iran, 2016

**Figure 2:**
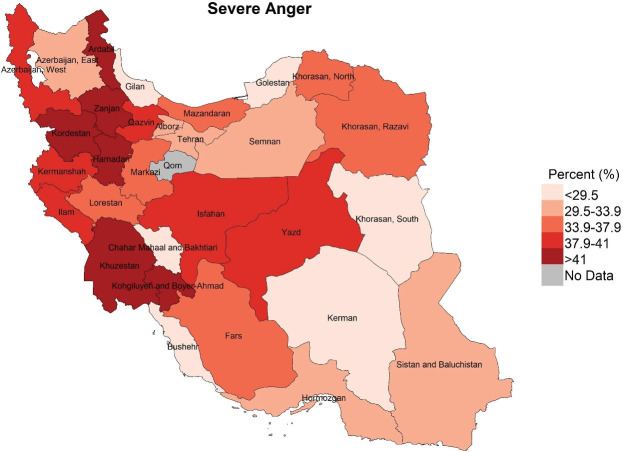
Distribution of Severe Anger among People Aged 18 Years Old and Over in the Provinces of Iran, 2016

**Figure 3:**
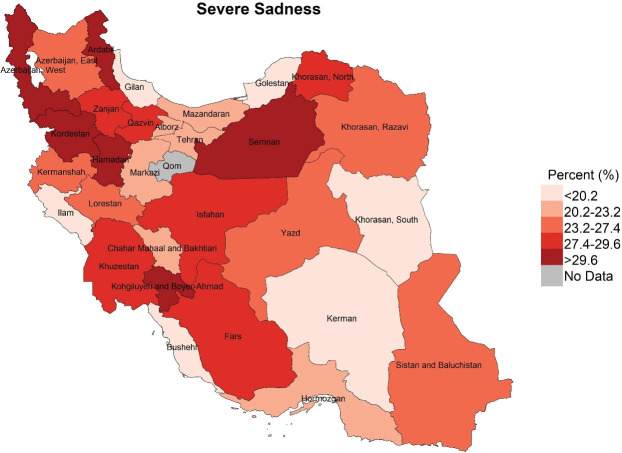
Distribution of Severe Sadness among People Aged 18 Years and older in the Provinces of Iran, 2016

 Descriptive statistics including frequency and percentage were used to describe the study variables (Table 1). Also, using the GIS software, three maps were made in order to visualize the distribution of different types of severe mental disorders in the provinces of Iran (Figures 1-3). Univariate and multivariate logistic regression analyses were used to examine the associations between the severe dimensions of mental disorders with the proposed determinants. Severe mental disorders were considered as outcome (dependent) variables and socio-demographic, lifestyle and health status factors were considered as exposure (independent) variables. In univariate analyses, the associations between every single exposure variable and outcome variables, once for all participants and again for men and women separately, were examined using logistic regression analyses (Table 2). To test the study hypotheses and examine the associations between exposure and outcome variables, four multivariate logistic regression models were run (Table 3). In Models 1, 2 and 3 the effects of each set of variables (socio-demographic, lifestyle and health status factors), on any of the outcome variables (types of severe mental disorders), adjusted for the effect of other variables in the same set of exposure variables, were tested. In Model 4, the effects of each exposure variable on any of the outcome variables, adjusted for the effects of all the other exposure variables in any set variables, were examined. All analyses were performed using the STATA software.

**Table 1:** Descriptive Statistics on Characteristics of Study Participants by Dimensions of Severe Mental Disorders and Gender

**Characteristics**	**Men**			**Women**			**All**		
	**Severe Stress** **N (%)**	**Severe Anger** **N (%)**	**Severe Sadness** **N (%)**	**Severe Stress** **N (%)**	**Severe Anger** **N (%)**	**Severe Sadness** **N (%)**	**Severe Stress** **N (%)**	**Severe Anger** **N (%)**	**Severe Sadness** **N (%)**
Gender	4176 (29.4)	4929 (34.3)	2962 (20.6)	5704 (36.4)	5647 (35.7)	4658 (29.7)	9880 (33.0)	10576 (35.3)	7620 (25.4)
Age									
18–24	349 (28.2)	444 (35.9)	263 (21.3)	497 (32.7)	481 (31.6)	356 (23.4)	846 (30.7)	925 (33.5)	619 (22.5)
25–34	1047 (31.3)	1185 (35.4)	689 (20.6)	1340 (35.3)	1376 (36.3)	1025 (27.0)	2387 (33.4)	2561 (35.9)	1714 (24.0)
35–44	1002 (32.7)	1153 (37.6)	646 (21.1)	1269 (38.5)	1300 (39.4)	1004 (30.5)	2271 (35.7)	2453 (38.6)	1650 (25.9)
45–54	778 (30.0)	890 (34.3)	532 (20.5)	1135 (39.3)	1106 (38.3)	932 (32.3)	1913 (34.9)	1996 (36.4)	1464 (26.7)
55–64	573 (27.9)	709 (34.5)	457 (22.2)	838 (37.5)	791 (35.5)	759 (34.0)	1411 (32.9)	1500 (35.0)	1216 (28.3)
65–70	148 (23.3)	182 (28.7)	126 (19.9)	283 (36.4)	259 (33.4)	229 (29.5)	431 (30.5)	441 (31.3)	355 (25.2)
Over 70	279 (20.9)	366 (27.4)	249 (18.6)	342 (28.8)	334 (28.1)	353 (29.7)	621 (24.6)	700 (27.7)	602 (23.9)
Wealth index									
Poorest	609 (22.9)	844 (31.8)	515 (19.4)	994 (31.0)	1100 (34.2)	933 (29.1)	1603 (27.3)	1944 (33.1)	1448 (24.7)
2	813 (29.9)	979 (36.0)	654 (24.1)	1159 (37.3)	1189 (38.2)	1033 (33.2)	1972 (33.9)	2168 (37.2)	1687 (29.0)
3	833 (29.5)	997 (35.3)	622 (22.0)	1150 (38.2)	1132 (37.7)	934 (31.0)	1983 (34.0)	2129 (36.5)	1556 (26.7)
4	863 (30.4)	1008 (35.5)	586 (20.7)	1144 (38.3)	1103 (37.0)	875 (29.3)	2007 (34.5)	2111 (36.3)	1461 (25.1)
Richest	939 (32.6)	1017 (35.3)	553 (19.2)	1085 (37.1)	1001 (34.2)	788 (26.9)	2024 (34.8)	2018 (34.7)	1341 (23.1)
Years of schooling									
0	333 (22.9)	468 (32.2)	323 (22.2)	1025 (32.9)	1115 (35.8)	1020 (32.74)	1358 (29.7)	1583 (34.7)	1343 (29.4)
1-6	877 (25.5)	1185 (34.5)	751 (21.9)	1521 (37.6)	1555 (38.4)	1328 (32.81)	2398 (32.0)	2740 (36.6)	2079 (27.8)
7-12	1952 (31.2)	2254 (36.0)	1312 (21.0)	2116 (37.5)	2026 (35.9)	1589 (28.16)	4068 (34.2)	4280 (36.0)	2901 (24.4)
> 12	1014 (32.5)	1022 (32.7)	576 (18.4)	1042 (36.2)	951 (33.0)	721 (25.0)	2056 (34.2)	1973 (32.9)	1297 (21.6)
Area									
Rural	1002 (24.0)	1384 (33.2)	849 (20.3)	1449 (31.0)	1669 (35.7)	1346 (28.8)	2451 (27.7)	3053 (34.5)	2195 (24.8)
Urban	3174 (31.4)	3545 (35.1)	2113 (20.9)	4255 (38.6)	3978 (36.1)	3312 (30.1)	7429 (35.2)	7523 (35.6)	5425 (25.7)
Marital status									
Never married	710 (29.3)	834 (34.4)	508 (21.0)	632 (32.2)	595 (30.4)	476 (24.3)	1342 (30.6)	1429 (32.6)	984 (22.5)
Married	3362 (29.2)	3977 (34.6)	2350 (20.4)	4172 (36.3)	4205 (36.5)	3320 (28.9)	7534 (32.7)	8182 (35.6)	5670 (24.6)
Divorced	46 (39.3)	48 (41.0)	40 (34.2)	209 (51.0)	195 (47.6)	196 (47.9)	255 (48.4)	243 (46.1)	236 (44.9)
Widow	39 (25.2)	46 (29.7)	46 (29.7)	658 (38.2)	626 (36.3)	633 (36.7)	697 (37.1)	672 (35.7)	679 (36.1)
Occupation									
Government employee	385 (31.6)	390 (32.1)	200 (16.4)	190 (40.5)	158 (33.6)	123 (26.3)	575 (34.1)	548 (32.5)	323 (19.2)
Government workers	85 (31.4)	91 (33.6)	42 (15.5)	12 (30.8)	11 (28.2)	9 (23.1)	97 (31.3)	102 (32.9)	51 (16.5)
Non-government employ	163 (32.4)	172 (34.3)	82 (16.3)	113 (40.8)	106 (38.3)	71 (25.6)	276 (35.4)	278 (35.7)	153 (19.6)
Non-government worker	418 (29.6)	504 (35.7)	285 (20.2)	52 (42.3)	51 (41.5)	38 (30.9)	470 (30.6)	555 (36.1)	323 (21.0)
Self-employee	1920 (29.3)	2387 (36.4)	1373 (20.9)	232 (42.2)	215 (39.1)	176 (31.9)	2152 (30.3)	2602 (36.6)	1549 (21.8)
Non-paid	16 (19.5)	20 (24.4)	16 (19.5)	33 (36.7)	25 (27.8)	27 (30.0)	49 (28.5)	45 (26.7)	43 (25.0)
Student	185 (29.7)	193 (30.9)	121 (19.4)	226 (36.5)	194 (31.3)	131 (21.1)	411 (33.0)	387 (31.1)	252 (20.3)
Soldier	29 (33.3)	34 (39.1)	18 (20.9)	—	—	—	29 (33.3)	34 (39.1)	18 (20.9)
Home keeper	46 (38.7)	41 (34.5)	37 (31.1)	4550 (36.1)	4640 (36.8)	3842 (30.5)	4596 (36.1)	4681 (36.8)	3879 (30.5)
Retired	489 (25.6)	560 (29.3)	388 (20.3)	106 (32.6)	81 (25.0)	89 (27.4)	595 (26.6)	641 (28.7)	477 (21.3)
Unemployed (unable to work because of health problems)	147 (24.5)	189 (31.5)	151 (25.1)	37 (27.8)	39 (29.3)	42 (31.6)	184 (25.1)	228 (31.1)	193 (26.3)
Unemployed (able to work but there is no job)	246 (34.3)	289 (40.3)	214 (29.9)	115 (34.6)	101 (30.4)	83 (25.0)	361 (34.4)	390 (37.2)	297 (28.3)
Unemployed (able to work but don’t want)	18 (26.5)	23 (33.8)	15 (22.1)	16 (42.1)	14 (36.8)	12 (31.6)	34 (32.1)	37 (34.9)	27 (25.5)
Insurance									
Health insurance	1449 (27.2)	1838 (34.5)	1187 (22.3)	2036 (34.7)	2095 (35.7)	1807 (30.8)	3485 (31.2)	3933 (35.1)	2994 (26.8)
Social insurance	1755 (30.0)	1983 (33.9)	1097 (18.7)	2443 (37.1)	2360 (35.8)	1827 (27.7)	4198 (33.7)	4343 (34.9)	2924 (23.5)
Army insurance	170 (29.5)	189 (32.8)	110 (19.1)	223 (35.6)	226 (36.0)	199 (31.7)	393 (32.7)	415 (34.5)	309 (25.7)
Imam Committee insurance	16 (32.0)	19 (38.0)	17 (34.0)	56 (41.8)	53 (39.6)	59 (44.0)	72 (39.1)	72 (39.1)	76 (41.3)
Other insurance	351 (30.1)	396 (34.0)	227 (19.5)	493 (34.9)	464 (32.9)	381 (27.0)	844 (32.8)	860 (33.4)	608 (23.6)
No insurance	433 (34.0)	497 (39.1)	320 (25.2)	443 (43.1)	444 (43.2)	376 (36.6)	876 (38.1)	941 (40.9)	696 (30.3)
Complimentary insurance									
No	3273 (29.1)	3965 (35.3)	2365 (21.0)	4393 (35.9)	4463 (36.5)	3638 (29.8)	7666 (32.7)	8428 (35.9)	6003 (25.6)
Yes	875 (29.9)	929 (31.8)	575 (19.7)	1253 (38.0)	1137 (34.5)	976 (29.6)	2128 (34.2)	2066 (33.2)	1551 (24.9)
Basic insurance									
No	433 (34.0)	497 (39.1)	320 (25.2)	443 (43.1)	444 (43.2)	376 (36.6)	876 (38.1)	941 (40.9)	696 (30.3)
Yes	3741 (28.8)	4425 (34.1)	2638 (20.3)	5251 (35.9)	5198 (35.6)	4273 (29.2)	8992 (32.6)	9623 (34.9)	6911 (25.0)
Income (Toman) in 2016									
< 700000	1508 (28.4)	1953 (36.8)	1294 (24.4)	2648 (37.1)	2754 (38.6)	2291 (32.1)	4156 (33.4)	4707 (37.8)	3585 (28.8)
700000–1500000	1695 (28.5)	2003 (33.7)	1132 (19.0)	1188 (37.7)	1155 (36.6)	926 (29.4)	2883 (31.7)	3158 (34.7)	2058 (22.6)
1500000–3000000	469 (32.2)	473 (32.4)	230 (15.7)	238 (40.4)	189 (32.1)	176 (29.9)	707 (34.5)	662 (32.3)	406 (19.8)
3000000–5000000	38 (32.2)	38 (32.2)	24 (20.3)	25 (56.8)	23 (52.3)	16 (36.4)	63 (38.9)	61 (37.7)	40 (24.7)
> 5000000	11 (34.4)	14 (43.8)	9 (28.1)	5 (45.5)	5 (45.5)	2 (18.2)	16 (37.2)	19 (44.2)	11 (25.6)
Tobacco smoking									
No	2931 (27.5)	3412 (32.0)	2005 (18.8)	5407 (35.9)	5333 (35.5)	4400 (29.3)	8338 (32.4)	8745 (34.0)	6405 (24.9)
Yes	1243 (34.8)	1513 (42.4)	953 (26.7)	289 (46.3)	306 (49.0)	251 (40.2)	1532 (36.5)	1819 (43.3)	1204 (28.7)
Cigarette consumption									
No	3195 (28.0)	3730 (32.7)	2199 (19.3)	5630 (36.3)	5570 (35.9)	4592 (29.6)	8825 (32.8)	9300 (34.5)	6791 (25.2)
Yes	979 (34.5)	1195 (42.1)	759 (26.8)	66 (45.8)	69 (47.9)	59 (41.0)	1045 (35.0)	1264 (42.4)	818 (27.4)
Serving at least 2 fruits in last 24 hours									
No	3360 (29.0)	3982 (34.4)	2456 (21.2)	4783 (36.5)	4787 (36.5)	3973 (30.3)	8143 (33.0)	8769 (35.5)	6429 (26.1)
Yes	803 (30.4)	923 (34.9)	491 (18.6)	898 (35.6)	837 (33.2)	660 (26.2)	1701 (32.9)	1760 (34.1)	1151 (22.3)
Serving vegetables in last 24 hours									
No	2513 (30.2)	3060 (36.8)	1884 (22.6)	3338 (37.5)	3385 (38.0)	2828 (31.8)	5851 (34.0)	6445 (37.4)	4712 (27.4)
Yes	1662 (28.1)	1864 (31.5)	1073 (18.1)	2358 (34.9)	2255 (33.4)	1822 (26.9)	4020 (31.7)	4119 (32.5)	2895 (22.8)
Low physical activity									
No	1862 (29.7)	2146 (34.2)	1251 (19.9)	2139 (38.8)	2117 (38.4)	1739 (31.5)	4001 (33.9)	4263 (36.2)	2990 (25.4)
Yes	1608 (29.1)	1848 (33.5)	1154 (20.9)	3342 (34.8)	3276 (34.1)	2700 (28.1)	4950 (32.7)	5124 (33.9)	3854 (25.5)
Hypertension									
No	3072 (28.8)	3642 (34.1)	2119 (19.9)	4030 (35.4)	4008 (35.2)	3207 (28.2)	7102 (32.2)	7650 (34.7)	5326 (24.2)
Yes	1093 (30.7)	1269 (35.6)	837 (23.5)	1661 (38.8)	1627 (38.0)	1440 (33.7)	2754 (35.1)	2896 (36.9)	2277 (29.1)
Diabetes									
No	2304 (28.2)	2815 (34.4)	1718 (21.0)	3433 (35.9)	3499 (36.6)	2880 (30.2)	5737 (32.3)	6314 (35.6)	4598 (25.9)
Yes	274 (33.0)	324 (39.1)	202 (24.4)	440 (38.1)	446 (38.7)	385 (33.4)	714 (36.0)	770 (38.9)	587 (29.6)
Diabetes HbA1c									
No	2284 (28.2)	2782 (34.4)	1697 (21.0)	3412 (36.0)	3483 (36.8)	2858 (30.2)	5696 (32.4)	6265 (35.7)	4555 (25.9)
Yes	294 (30.8)	370 (38.8)	223 (23.4)	489 (38.1)	487 (38.0)	425 (33.2)	783 (35.0)	857 (38.3)	648 (29.0)
Cholesterol									
No	2074 (28.1)	2538 (34.3)	1558 (21.1)	2878 (35.6)	2952 (36.5)	2396 (29.6)	4952 (32.0)	5490 (35.5)	3954 (25.5)
Yes	512 (30.7)	613 (36.8)	367 (22.0)	1026 (38.2)	1024 (38.1)	890 (33.1)	1538 (35.3)	1637 (37.6)	1257 (28.9)
Heart attack since last year									
No	4081 (29.2)	4817 (34.4)	2884 (20.6)	5609 (36.2)	5555 (35.9)	4569 (29.5)	9690 (32.9)	10372 (35.2)	7453 (25.3)
Yes	92 (35.1)	108 (41.2)	75 (28.6)	90 (50.6)	88 (49.2)	83 (46.4)	182 (41.4)	196 (44.4)	158 (35.8)
Ischemic stroke since last year									
No	4136 (29.2)	4878 (34.5)	2921 (20.7)	5649 (36.3)	5593 (35.9)	4607 (29.6)	9785 (32.9)	10471 (35.2)	7528 (25.3)
Yes	37 (34.3)	47 (43.5)	38 (35.2)	53 (49.5)	52 (48.6)	47 (43.9)	90 (41.9)	99 (46.1)	85 (39.5)
All Participants	—	—	—	—	—	—	29964 (100%)	29962 (100%)	29965 (100%)

**Table 2:** Univariate Logistic Regression Analysis on the Associations Between Characteristics of Participants and Dimensions of Severe Mental Disorders

**Characteristics** **OR (** ***P*** **)**	**Men**			**Women**			**All**		
	**Severe Stress**	**Severe Anger**	**Severe Sadness**	**Severe Stress**	**Severe Anger**	**Severe Sadness**	**Severe Stress**	**Severe Anger**	**Severe Sadness**
Gender									
Women	Ref.	Ref.	Ref.	Ref.	Ref.	Ref.	Ref.	Ref.	Ref.
Men	—	—	—	—	—	—	0.73***	0.94	0.62***
Age	0.99 ***	0.99***	1.00	0.99	1.00**	1.01***	0.99**	1.00**	1.00**
Age group									
18–24	Ref.	Ref.	Ref.	Ref.	Ref.	Ref.	Ref.	Ref.	Ref.
25–34	1.16	0.99	1.00	1.16*	1.24**	1.21*	1.16**	1.12*	1.11
35–44	1.26**	1.08	1.03	1.30***	1.41***	1.43***	1.28***	1.24***	1.23***
45–54	1.11	0.94	0.98	1.33***	1.34***	1.53***	1.22***	1.13*	1.26***
55–64	1.00	0.96	1.10	1.24**	1.18*	1.65***	1.12*	1.07	1.38***
65–70	0.77*	0.71**	0.91	1.19	1.08	1.36**	1.00	0.90	1.16
Over	0.69***	0.67***	0.85	0.85	0.85	1.37***	0.76***	0.76***	1.08
Wealth index									
Poorest	Ref.	Ref.	Ref.	Ref.	Ref.	Ref.	Ref.	Ref.	Ref.
2	1.49***	1.24***	1.39***	1.33***	1.20**	1.21**	1.39***	1.21***	1.27***
3	1.41***	1.18**	1.20**	1.38***	1.18**	1.10	1.37***	1.18***	1.12**
4	1.48***	1.19**	1.10	1.37***	1.13*	1.01	1.40***	1.16***	1.03
Richest	1.59***	1.17**	0.98	1.28***	1.00	0.88*	1.39***	1.07	0.90*
Years of schooling									
0	Ref.	Ref.	Ref.	Ref.	Ref.	Ref.	Ref.	Ref.	Ref.
1–6	1.20*	1.11	1.01	1.21***	1.12*	1.00	1.12**	1.09*	0.93
7–12	1.56***	1.19**	0.96	1.21***	1.00	0.80***	1.22***	1.06	0.78***
> 12	1.60***	1.01	0.80**	1.14*	0.88*	0.68***	1.20***	0.91*	0.66***
Area									
Rural	Ref.	Ref.	Ref.	Ref.	Ref.	Ref.	Ref.	Ref.	Ref.
Urban	1.44***	1.10*	1.05	1.38***	1.02	1.05	1.40***	1.05	1.05
Marital status									
Never married	Ref.	Ref.	Ref.	Ref.	Ref.	Ref.	Ref.	Ref.	Ref.
Married	1.01	1.03	0.97	1.16**	1.30***	1.24***	1.10*	1.14***	1.12**
Divorced	1.56*	1.35	1.92**	2.12***	2.09***	2.81***	2.09***	1.79***	2.79***
Widowed	0.80	0.79	1.54*	1.27**	1.29***	1.77***	1.33***	1.15*	1.94***
Occupation									
Government employee	Ref.	Ref.	Ref.	Ref.	Ref.	Ref.	Ref.	Ref.	Ref.
Government workers	0.99	1.02	0.87	0.68	0.81	0.93	0.88	0.98	0.79
Non-government employ	1.05	1.11	0.95	1.02	1.26	0.99	1.08	1.17	1.01
Non-government worker	0.96	1.20*	1.35**	1.14	1.47	1.31	0.90	1.20*	1.18
Self-employee	0.93	1.25**	1.37***	1.04	1.28	1.36*	0.86*	1.24***	1.20*
Non-paid	0.51*	0.66	1.05	0.81	0.78	1.18	0.73	0.74	1.29
Student	0.94	0.94	1.18	0.85	0.93	0.77	0.97	0.95	1.07
Soldier	0.93	1.25	1.11	-	-	-	0.82	1.22	0.92
Home keeper	1.51*	1.25	2.47***	0.82*	1.16	1.24*	1.11	1.23***	1.87***
Retired	0.76**	0.90	1.31**	0.70*	0.65***	1.04	0.71***	0.85*	1.15
Unemployed (unable to work because of health)	0.74*	1.00	1.73***	0.56**	0.82	1.24	0.67***	0.96	1.50***
Unemployed (able to work but there is no job)	1.10	1.42***	2.14***	0.77	0.87	0.92	0.99	1.23	1.64***
Unemployed (able to work but don’t want)	0.74	1.06	1.25	1.10	1.25	1.29	0.90	1.13*	1.32
Insurance									
Health insurance	Ref.	Ref.	Ref.	Ref.	Ref.	Ref.	Ref.	Ref.	Ref.
Social insurance	1.13**	0.97	0.80***	1.09*	1.00	0.86***	1.11***	0.98	0.84***
Army insurance	1.10	0.92	0.79*	1.01	0.99	1.06	1.05	0.96	0.94
Imam Committee insurance	1.21	1.21	1.77	1.25	1.15	1.67**	1.33	1.18	1.85***
Other insurance	1.14	0.95	0.84*	0.96	0.85*	0.80**	1.04	0.89*	0.83***
No insurance	1.40***	1.19*	1.20*	1.39***	1.34***	1.29***	1.35***	1.25***	1.20**
Complimentary insurance									
No	Ref.	Ref.	Ref.	Ref.	Ref.	Ref.	Ref.	Ref.	Ref.
Yes	1.02	0.85***	0.89*	1.07	0.92*	0.97	1.05	0.88***	0.95
Basic insurance									
No	Ref.	Ref.	Ref.	Ref.	Ref.	Ref.	Ref.	Ref.	Ref.
Yes	0.77***	0.82**	0.74**	0.75***	0.74***	0.72***	0.78***	0.79***	0.76***
Income (Toman) in 2016									
< 700000	Ref.	Ref.	Ref.	Ref.	Ref.	Ref.	Ref.	Ref.	Ref.
700000–1500000	0.98	0.87***	0.72***	1.02	0.91*	0.87**	0.91**	0.87***	0.71***
1500000–3000000	1.14*	0.80**	0.56***	1.11	0.74**	0.86	1.01	0.77***	0.59***
3000000–5000000	1.13	0.76	0.71	2.11*	1.67	1.24	1.18	0.92	0.75
> 5000000	1.03	1.10	1.05	1.28	1.22	0.52	0.97	1.11	0.78
Tobacco smoking									
No	Ref.	Ref.	Ref.	Ref.	Ref.	Ref.	Ref.	Ref.	Ref.
Yes	1.39***	1.57***	1.55***	1.53***	1.74***	1.60***	1.19***	1.49***	1.20***
Cigarette consumption									
No	Ref.	Ref.	Ref.	Ref.	Ref.	Ref.	Ref.	Ref.	Ref.
Yes	1.35***	1.52***	1.51***	1.46*	1.59**	1.59***	1.10*	1.41***	1.10*
Serving 2 fruits in last 24 h									
No	Ref.	Ref.	Ref.	Ref.	Ref.	Ref.	Ref.	Ref.	Ref.
Yes	1.06	1.02	0.84**	0.95	0.87**	0.81***	0.99	0.94*	0.81***
Serving vegetables in last 24 h									
No	Ref.	Ref.	Ref.	Ref.	Ref.	Ref.	Ref.	Ref.	Ref.
Yes	0.89**	0.78***	0.75***	0.89**	0.81***	0.78***	0.89***	0.79***	0.77***
Low physical activity									
No	Ref.	Ref.	Ref.	Ref.	Ref.	Ref.	Ref.	Ref.	Ref.
Yes	0.99	0.99	1.09	0.84***	0.83***	0.85***	0.95	0.91***	1.02***
Hypertension									
No	Ref.	Ref.	Ref.	Ref.	Ref.	Ref.	Ref.	Ref.	Ref.
Yes	1.11*	1.06	1.26***	1.15***	1.13**	1.29***	1.14***	1.10**	1.29*
Diabetes (fasting plasma glucose)									
No	Ref.	Ref.	Ref.	Ref.	Ref.	Ref.	Ref.	Ref.	Ref.
Yes	1.37**	1.32**	1.24*	1.03	1.04	1.10	1.17*	1.15*	1.17
Diabetes (HbA1c)									
No	Ref.	Ref.	Ref.	Ref.	Ref.	Ref.	Ref.	Ref.	Ref.
Yes	1.22*	1.33**	1.16	1.03	1.02	1.08	1.11	1.14*	1.13*
Cholesterol									
No	Ref.	Ref.	Ref.	Ref.	Ref.	Ref.	Ref.	Ref.	Ref.
Yes	1.16*	1.10	1.07	1.14*	1.10	1.16*	1.18***	1.11*	1.17**
Heart attack since last year									
No	Ref.	Ref.	Ref.	Ref.	Ref.	Ref.	Ref.	Ref.	Ref.
Yes	1.34*	1.38*	1.55**	1.72***	1.66**	2.07***	1.43***	1.48***	1.66***
Ischemic stroke since last year									
No	Ref.	Ref.	Ref.	Ref.	Ref.	Ref.	Ref.	Ref.	Ref.
Yes	1.24	1.41	2.09***	1.77**	1.74**	1.82**	1.47**	1.56**	1.90***

OR, Odds Ratio.* P*, *P* value (*P < 0.05; **P < 0.01; ***P < 0.001).

**Table 3:** Models of Multivariate Logistic Regression Analysis on the Associations Between Characteristics of Participants and Dimensions of Severe Mental Disorders

**Characteristics** **OR (** ***P*** **)**	**Severe Stress**				**Severe Anger**				**Severe Sadness**			
	**Model 1**	**Model 2**	**Model 3**	**Model 4**	**Model 1**	**Model 2**	**Model 3**	**Model 4**	**Model 1**	**Model 2**	**Model 3**	**Model 4**
Gender												
Women	Ref.	—	—	Ref.	Ref.	—	—	Ref.	Ref.	—	—	Ref.
Men	0.71***	—	—	0.66***	0.96	—	—	0.88*	0.65***	—	—	0.57***
Age	0.99***	—	—	0.99***	0.99***	—	—	0.98***	1.00	—	—	0.99**
Wealth index												
Poorest	Ref.	—	—	Ref.	Ref.	—	—	Ref.	Ref.	—	—	Ref.
2	1.30***	—	—	1.29***	1.21***	—	—	1.27***	1.28***	—	—	1.28**
3	1.25***	—	—	1.28**	1.16**	—	—	1.29***	1.18**	—	—	1.18*
4	1.26***	—	—	1.35***	1.15**	—	—	1.30**	1.13*	—	—	1.20*
Richest	1.23***	—	—	1.14	1.14*	—	—	1.16	1.06	—	—	0.97
Years of schooling												
0	Ref.	—	—	Ref.	Ref.	—	—	Ref.	Ref.	—	—	Ref.
1–6	1.06	—	—	1.12	0.96	—	—	0.97	1.05	—	—	1.11
7–12	1.08	—	—	1.15	0.89*	—	—	0.91	0.92	—	—	0.98
> 12	1.09	—	—	1.24*	0.81**	—	—	0.96	0.85*	—	—	0.95
Area												
Rural	Ref.	—	—	Ref.	Ref.	—	—	Ref.	Ref.	—	—	Ref.
Urban	1.29***	—	—	1.33***	1.08*	—	—	1.08	1.11*	—	—	1.15**
Marital status												
Never married	Ref.	—	—	Ref.	Ref.	—	—	Ref.	Ref.	—	—	Ref.
Married	1.25***	—	—	1.30**	1.35***	—	—	1.46***	1.16**	—	—	1.10
Divorced	2.04***	—	—	1.72**	1.95***	—	—	1.63**	2.14***	—	—	1.85**
Widowed	1.63***	—	—	1.85***	1.56***	—	—	1.68***	1.62***	—	—	1.45**
Basic insurance												
No	Ref.	—	—	Ref.	Ref.	—	—	Ref.	Ref.	—	—	Ref.
Yes	0.79***	—	—	0.84	0.80***	—	—	0.84	0.77***	—	—	0.81
Income (Toman) in 2016												
< 700000	Ref.	—	—	Ref.	Ref.	—	—	Ref.	Ref.	—	—	Ref.
700000–1500000	0.91**	—	—	0.89*	0.88***	—	—	0.83***	0.80***	—	—	0.85**
1500000–3000000	1.00	—	—	0.94	0.81***	—	—	0.77**	0.72***	—	—	0.84
3000000–5000000	1.18	—	—	1.40	0.99	—	—	0.81	0.96	—	—	1.56
> 5000000	0.98	—	—	0.53	1.23	—	—	0.47	1.00	—	—	0.47
Tobacco smoking												
No	—	Ref.	—	Ref.	—	Ref.	—	Ref.	—	Ref.	—	Ref.
Yes	—	1.22***	—	1.47***	—	1.51***	—	1.49***	—	1.19***	—	1.43***
Serving 2 fruits in last 24 h												
No	—	Ref.	—	Ref.	—	Ref.	—	Ref.	—	Ref.	—	Ref.
Yes	—	1.03	—	1.01	—	1.02	—	1.03	—	0.88**	—	0.93
Serving vegetables in last 24 h												
No	—	Ref.	—	Ref.	—	Ref.	—	Ref.	—	Ref.	—	Ref.
Yes	—	0.90***	—	0.83***	—	0.81***	—	0.77***	—	0.81***	—	0.83***
Low physical activity												
No	—	Ref.	—	Ref.	—	Ref.	—	Ref.	—	Ref.	—	Ref.
Yes	—	0.95	—	0.87**	—	0.91***	—	0.88**	—	1.01***	—	0.85**
Hypertension												
No	—	—	Ref	Ref	—	—	Ref	Ref	—	—	Ref	Ref
Yes	—	—	1.04	1.20**	—	—	1.04	1.23***	—	—	1.15**	1.16*
Diabetes												
No	—	—	Ref	Ref	—	—	Ref	Ref	—	—	Ref	Ref
Yes	—	—	1.11	1.22*	—	—	1.10	1.29**	—	—	1.09	1.10
Cholesterol												
No	—	—	Ref	Ref	—	—	Ref	Ref	—	—	Ref	Ref
Yes	—	—	1.15**	1.21***	—	—	1.07	1.16**	—	—	1.12*	1.15*
Heart attack since last year												
No	—	—	Ref	Ref	—	—	Ref	Ref	—	—	Ref	Ref
Yes	—	—	1.27	1.48*	—	—	1.34*	1.56**	—	—	1.14	1.34
Ischemic stroke since last year												
No	—	—	Ref	Ref	—	—	Ref	Ref	—	—	Ref	Ref
Yes	—	—	1.07	1.12	—	—	1.42	1.59	—	—	1.50	1.35

OR, Odds Ratio; *P*, *P* value (*P < 0.05; **P < 0.01; ***P < 0.001). In model 1, only socio-demographic variables were adjusted. In model 2, only lifestyle variables were adjusted. In model 3, only health status variables were adjusted. In model 4, all thevariables were adjusted at each dimension of mental disorders.

## Results

###  Descriptive Findings

 According to Table 1, of the total 30 541 people participating in this study, 29964, 29962, and 29965 people answered to severe stress, sever anger, and severe sadness questions, respectively (average response rate = 98%). Of these, 9880 people (33%) experienced severe stress, 10576 people (35%) severe anger, and 7620 people (25%) severe sadness. Among these respondents, the percentage of women who experienced each of these disorders was higher than men. Thus, 36.4% of women versus 29.4%, 35.7% of women versus 34.3% of men, and 29.7% of women versus 20.6% of men experienced severe stress, severe anger and severe sadness, respectively.

 Among all age groups, the percentage of people who experienced severe anger was higher than those who experienced severe stress and sadness. People over the age of 70 experienced less stress, anger and sadness than other age groups. In the case of the wealth index, in all categories except the richest people, the percentage of people who experienced extreme anger was higher than those who experienced sadness and stress. However, in these people, the percentage of those who experienced severe stress was higher than the other two cases. In terms of school years, people who studied for less than 12 years, experienced more intense anger than sadness and stress, but people with more than 12 years of schooling experienced more severe stress. In addition, in terms of place of residence, both for women and men, the proportion of exposed people in urban areas in all the three types of severe mental disorders were higher than that in rural dwellers.

 Among all individuals, whether single, married, divorced or widowed, severe sadness was less than the other two disorders. Single women had less anger, but more sadness and stress than single men. In married women, widows and divorced, all three disorders were more common than men. All three disorders were less common in singles. In terms of employment status, severe sadness were less than the other two disorders. People with an income less than 700 thousand Toman had higher sadness, group with an income more than 5 million Toman, had higher severe anger and in the middle group severe stress was higher than the other two groups.

 With regard to the lifestyle factors, all three disorders were more common smokers and those with low consumption of fruits and vegetables than non-smokers and people with suitable diet. However, people with less physical activity had lower stress and anger and same level of sadness compared others. Additionally, all the three types of mental disorders were more common among NCDs patients than healthy people.

 Mapping of distribution of the three severe mental disorders among the provinces of the country, shown in Figure 3 indicates that the experience of severe stress was higher in Ardabil, Kermanshah, Qazvin, Tehran and Isfahan, Kohkiluyeh and Boyer-Ahmad than the other provinces (more than 35.8%). Severe anger was also more than 41% in Kohkiluyeh and Boyer-Ahmad, Khuzestan, Hamedan, Kurdistan, Zanjan and Ardabil. Additionally, in the provinces of Ardabil, West Azerbaijan, Kurdistan, Hamedan, Semnan, and Kohkiluyeh and Boyer-Ahmad, more than 29.6% experienced severe sadness. Thus, the maps highlights that Ardabil, Kohkiluyeh and Boyer-Ahmad were among the provinces in which all the three were considerably more common. In contrast, South Khorasan, Bushehr, Kerman, Gilan and Golestan were among the provinces in which all the three disorders were less prevalent.

###  Associations Between Individual Characteristics and Severe Mental Disorders 

 Associations between various sets of factors and outcome variables were tested using univariate and multivariate regression analyses and results are shown in Tables 2 and 3 respectively. As multivariate models have adjusted for the effects of all the other exposure variables, are more worthy for attention and constitutes most of the results reported here.

 As shown in Table 2 and 3, in univariate regression analysis, gender was significantly associated with severe stress and sadness, and men experienced severe stress and severe sadness more than women, respectively. In multivariate regression analysis, all the three disorders had a significant relationship with gender and this relationship was stronger in terms of anger. Men experienced 0.66, 0.88 and 0.57 times less stress, anger and sadness compared to women, respectively (Table 3). Age also showed strong associations with all the three mental disorders in multivariate regression analysis; increasing age was strongly associated with lower experience of severe stress, anger and sadness. With regard to the associations of wealth index and mental disorders, multivariate analyses (Table 3) showed that with improving the socioeconomic status of people, they also experience higher levels of severe mental disorders compared to the poorest group. Only exception is for the richest group, where there was no significant difference between them and the poorest group, but in model 1 they still experience more stress and anger compared to the poorest group.

 Increasing years of schooling, generally speaking, showed little associations with severe mental disorders in multivariate analyses (Table 3). Only people in the group of > 12 years of schooling, experienced significantly higher stress (model 4), but lower anger (model 1) and lower sadness (model 1), compared to the illiterate group. Groups with lower education (less than12 years) were identical compared to the illiterate people, with regard to their experience of severe mental disorders. Place of residence, was another factor of investigation for its association with mental disorders. Results of univariate analyses in Table 2, showed that those living in urban areas, both men and women, experienced strongly higher stress compared to people of rural areas, but almost same level of anger and sadness. However, in multivariate analysis, strong associations were found between this factor and all types of disorders, and urban dwellers experienced strongly higher stress, sadness and anger, except model 4 in sever anger, than the rural dwellers (Table 3).

 Analyses for associations of marital status with experiencing severe mental disorders in Table 2 showed that all people and women in the groups of married, divorced, and widowed were strongly more likely to report sever stress, anger and sadness compared to never married group. However, only divorced men had higher reporting of stress and sadness compared to single men. In multivariate analyses (Table 3), marital status showed very strong associations with mental disorders in all the three types and married, divorced, and widowed people reported strongly higher experience of disorders compared to never married adults.

 Having health insurance also should to be associated with lower experience of severe stress, anger and sadness in models 1, compared to lacking insurance. However, in models 4 no significant difference was found among two groups. Income level also showed some relationships with mental disorders, but mostly among the first group with lowest income (less than 700 thousand Toman) and the second group (700 thousand - 1.5 million Toman) in severe stress and also with the next two better groups in sever anger and sadness. But no difference there was between the richest group and poorest group in any of the three disorders. (Table 3)

###  Associations Between Lifestyle and Severe Mental Disorders 

 Using univariate analyses (Table 2), it was found that tobacco and cigarette smoking both were strongly associated with all the three types of mental disorders among men, women and all participants. In multivariate analysis, this relationship was stronger in models 1 and 4. Smokers were 1.47, 1.49 and 1.43 times more likely to experience severe stress, anger and sadness than non-smokers, respectively (Table 3). Regarding fruit consumption in univariate analysis, there was less chance of experiencing anger and sadness among those serving at least 2 fruits, but in model 2 of multivariate analysis, the relationship was only remained for severe sadness. Consumption of vegetables showed to be more important than fruit for prevention of getting severe mental disorders among all group in Table 2. In multivariate analysis, also consumption of vegetables demonstrated a significant relationship, in both models 1 and 4 and there was 0.83, 0.77 and 0.83 times less likely to get severe stress, anger, and sadness, respectively, compared to when people do not use vegetables. In the case of low physical activity, univariate analysis did not show an association with severe stress but it was associated with lower sadness and anger. In multivariate analysis, there was no relationship with stress in model 1, but in model 4, a significant relationship was found. The other two disorders had a significant relationship in both models 1 and 4 and the relationship was persisted, so that people who had low physical activity also had less experience of severe stress, anger and sadness 0.87, 0.88 and 0.85 times, respectively, compared to those who do exercise.

###  Associations between Non-communicable Diseases and Severe Mental Disorders 

 Considering the results of the associations between having different NCDs and at the same time experiencing severe mental disorders in Tables 2 and 3 indicated that these two factors are almost highly correlated with each other. With regard to the hypertension, multivariate analysis (model 4) showed that all three disorders are associated with higher experience of severe stress, anger and sadness about 1.20, 1.23 and 1.16 times more compared to healthy people. Similarly, suffering from diabetes reported 1.22 and 1.29 times more likely to get severe stress and anger than the others in multivariate analysis (model 4). In the case of high cholesterol, the findings are the same; patients experienced 1.21, 1.16, and 1.15 times more stress, anger, and sadness than the others. Also patients with heart attack experienced significantly more stress and anger, 1.48 and 1.56 times higher than those with no such problem. However, in the case of getting ischemic stroke since last year, no significant relationship was found with any of the three disorders in multivariate analysis.

## Discussion

 The results of this study indicate that the prevalence of severe stress, severe anger and sever sadness in the Iranian society is 33%, 35%, and 25%, respectively while according to results of a meta-analysis study in the world in 2015, the prevalence of mental disorders in the world is on average 13.4%, indicating a considerably higher rate of the problem in this context.^26^ The present study aimed to examine the relationship between severe mental disorders with a list of socio-demographic, lifestyle and health status factors. Globally, there is numerous evidence that mental and psychiatric disorders could be mainly affected by general health status, demographical and socio-economic factors,^10-13^ however these associations were less understood with regard to severe mental problems. According to the analyses, gender was associated with all the three disorders and women experienced more severe stress, anger and sadness than men, in particular severe anger. The result of a systematic review and meta-analysis in Iran showed that females were about twice more likely to have a major depressive disorder.^27^ In addition, rich people experienced more stress and anger than poor people. According to a study by Daniel Kahneman, high income brings life satisfaction but does not necessarily lead to happiness, and low income is associated with both low life evaluation and low emotional well-being.^28^ The results showed that older people experienced less stress, anger and sadness. As Schieman points out, age of people effects on their roles, their personal and social circumstances, sense of control, health, and socio-emotional outlook.^29^ On the other hand, with increasing age, a person’s life experiences in dealing with different situations differs and persons will be better able to control situations which prevents stress, anger or sadness.

 With the number of years of schooling exceeding 12 years, the severity of all three disorders increased. It can be said that with increasing level of education, people may be more stressed to achieve better jobs and social positions. Especially in societies where there is little opportunity to use the academic ability and talent of people, the stress will be greater. According to studies, stress is the basis for other emotional reactions such as sadness and anger.^30^ Therefore, with increasing education, stress, sadness and anger might be expected to be increased too. Also, urban dwellers experience more intense stress and sadness than rural residents. In the line of our findings, urban life was associated with mental disorders in the study by Sharifi et al.^8^ However, inconsistently the study by Sadeghirad et al showed that place of resident was not recognized as a significant factor.^27^

 Married, divorced, and widowed individuals experienced more stress and anger than single people. Divorced and widowed people also experienced more sadness than single people. These findings are in line with that of Sharifi et al^8^ who examined mood, anxiety, and substance use mental disorders in 7886 individuals, and found that being widowed/divorced was associated with a greater likelihood of psychiatric disorders, while higher socioeconomic status and having a university degree were associated with a lower likelihood. Generally, in Iran, there is some evidence indicating that age, marital status, education, family income, occupation, region of residence, inequality, individual health conditions are the main contributors to the mental health status of populations.^6,15,16^

 Surprisingly, people with low physical activity, particularly women, experienced less severe disorders, while, on the other hand, hypertension and high cholesterol levels were associated with more stress, anger and sadness than other people. Most of the existing evidence, contrarily, demonstrated that better mental health was independently associated with physical activity, and emphasizes the importance of obesity and daily smoking for poor mental health.^31,32^ Also, a Callaghan literature review study puts emphasis on exercise as a beneficial intervention for mental health care.^33^ In line with this, the study by Kiessling et al claims that in adolescents dealing with hypertension, one certainly wonders if undiagnosed and non-treated mental health problems, including anxiety and depression in an asymptomatic individual, can lead to medical non-compliance and worsen hypertension and related end organ disease.^34^ Therefore, these results from Iran actually needs further investigations in further studies. In addition, people with a history of heart attack also have experienced more stress and anger than others. This finding has been confirmed in various studies.^35,36^

 As mentioned previously, compared to other studies that used household income to measure economic status, this study has the advantage of measuring economic status using wealth index, which is a more accurate index. This method has fewer limitations compared to direct measure in developing countries.^14,22^ In addition, instead of applying linear regression to decompose inequality in a non-linear setting, the current paper used a more appropriate method to accomplish its objective. Nevertheless, our study also has a number of limitations. Firstly, the measurement of severe mental disorders in this study is based on only three single questions; while the single item questions have obvious benefits for both research and policy in terms of reduced burden and costs, and ease of interpretation, and can be reliable and valid, it is at the expense of details.^37^ More information may be required on different dimensions of server mental health than a single item can provide. Also, this study is one of the limited studies that considers the severe status of mental health in relation to main determinants. However, given that these determinants were selected based on availability on this survey, some of the important variables may not have been considered. In addition, our data were drawn from a cross-sectional study; attribution of causal interpretations to the results should be done with caution. In fact, longitudinal data are necessary here. Furthermore, because our study only covers subjects aged 18 and over, we have no information on the distribution of mental health in children and juveniles, who make up a very significant proportion of the Iranian population. Another limitation of our study is slightly higher proportion of women in the analysis (52.31% female). Our intention was to have approximately equal numbers of women and men in the study, in accordance with Tehran’s population structure; however, when the GHQ-28 was administered, the available member of the household was interviewed, who was a woman in most cases. This might have affected the results in a number of ways. Indeed, some of the socioeconomic inequality in mental health could be due to this issue. This should be borne in mind when interpreting our findings.

 In conclusion, this study showed that some main socio-economic factors including age, education, income level, wealth quantile, marital status, and occupation, lifestyle factors, and health status contribute in experiencing severe mental disorders. By considering these determinants, it can be concluded that raising awareness of people; improving lifestyle by encouraging exercise, leaving smoke, and consuming enough vegetables and fruits; and screening high risk people can lead to a reduction in mental disorders. The results of this study can provide a broad knowledge on mental disorders determinants for policy makers and researchers in order to reduce the complications and burden caused by these disorders. Further studies are required in order to recognize the effects of these determinants on other dimensions of mental health.
